# Texas Goes Foot—Defeat of the Vasectomy Bill

**Published:** 1913-04

**Authors:** 


### EDITORIAL DEPARTMENT.

DR. F. E. DANIEL, Editor DR. R. H. L. BIBB, Associate Editor

DEPARTMENTS { EUGENICS: Drs. M. Duggan and T. Y. Hull.

WOMAN'S DEPARTMENT: Mrs. F. E. Daniel.

### Texas Goes Foot—Defeat of the Vasectomy Bill.

The Texas Senate, on March 29, by a vote of 14 to 11, 6 absent or paired, killed the Parker bill for the sterilization of the defectives in the State institutions.

It was not through opposition to the bill, but because of ignorance of it.

It is most remarkable that some of those sent here, supposedly representing the intelligence and honesty of their constituency, should not only fail to make themselves acquainted with the provisions of this, the most important politico-economic and humanitarian reform measure of the century, but that they should, by reason of that neglect, misrepresent it before the Senate and put it in a false light, thus defeating it. They read into it, as Judge White of the United States Supreme Court did into the celebrated anti-trust case, something that was not in it, something entirely foreign to its aims, something which appealed to the prejudices of the average member. To be specific, Senator Morrow opened the opposition by declaring that the proposed operation was "cruel and unusual punishment" and therefore unconstitutional, and he made his play entirely on this one string. If he had even read the bill he would have seen that the little operation is not proposed as a *punishment* for *anything,* and the word "punishment" does not occur in the bill. If he had been honest and fair he would not have so misrepresented the bill. By his effective misrepresentation he has sanctioned the continued flow of degenerates by hereditary descent from those whose care and maintenance is costing the taxpayers millions yearly and increasing in a ratio of geometric progression. The Texas State Medical Association and the superintendents of our four asylums have realized the necessity of some effort to arrest this deluge of idiots, imbeciles, lunatics, epileptics and hereditary and confirmed criminals, and have asked for the passage of a bill of the kind. The more enlightened lawmakers in ten or more States have seen the wisdom and necessity of such law, and it is now in operation from California through the West and into New York, New Jersey and Connecticut, and probably by now in the more Western States of Michigan, Minnesota and Kansas. Hence I say, *Texas goes foot.*

In the discussion—if I can so dignify the senseless talk of certain Senators—one gentleman drew a pathetic picture of a young couple who had built a nest and looked forward to the laugh of many children; pictured their sadness and sorrow and disappointment because some "board," he said, had sterilized hubby. The idiot! The bill confined the operation to the inmates of the charity institutions and prisons—to the wreckage of humanity—to those whose lives had been wrecked by their ancestors. Did he suppose that "boards" were going about seeking whom they might sterilize? Hubby was most likely sterilized by gonorrheal orchitis.

And Kauffman—the doctor's friend—thought that the confirmed criminals would reform—spontaneously! Well, Kauffman's father and grandfather were preachers, and I suppose he had inherited that blessed hope he had doubtless heard them sing about—the lamp and the vile sinner. And a fellow—beg pardon, a Senator—named Westbrook tried to be funny. He said, "The medical fraternity has already usurped" (yes, "usurped," he said) "more public functions than is good for the general welfare," and he attempted some jokes. But here comes in the real burlesque—for the performance was (unintentionally) better than a circus. A big fellow—beg pardon, a Senator—named Brelsford, with a voice like a megaphone, made the hall resound with pleas—almost tearful—for "the God-given right of procreation." He thought the bill immoral and indefensible, and wanted to know if we were going to put mankind on a level with the cattle. He suggested that some day a cure might be found for the ailments and defects for which the breeding of these people was to be prevented—that is, for idiocy, imbecility, epilepsy, feeble-mindedness, lunacy and crime. I'll suggest it to Simon Flexner and recommend a consultation with Mr. Brelsford. He may have something to offer in that line.

Nevertheless, this stupid action killed the bill. It was Carlisle who said that ignorance and stupidity are a stronger force in the world than education and culture. They certainly are, when in power. This means more asylums, jails, poorhouses and reformatories and homes for feeble-minded. But it means more. The next generation is to be burdened by their increase, and so on for ages. We have to care for those already on hand, but it certainly is the part of wisdom, statesmanship and common sense to prevent the increase. Suppose Max Jukes had been sterilized? Record of 1200 of his progeny shows all degenerate.

These fourteen learned (?) Senators killed the bill with their eyes and ears shut to the fact that the insane of Texas are costing the people a million a year, and the criminals as much more. They did this in knowledge of the fact that the asylums are now asking this Legislature for nearly a million more to provide more room for more insane and epileptics, and that the Legislature has just authorized the expenditure of $75,000 for a home for feeble-minded (vetoed by Colquitt), and an issue of $2,000,000 bonds for the penitentiary. Why argue with such men, when they shut their eyes to such facts?

It were profitless to enumerate the reasons why such law should be enacted. The defectives are increasing faster than provision can be made for their care. A proposition to avert this increase by a perfectly harmless and humane process would seem to need no argument, but we have with us still some Brother Jaspers who insist that "the sun do move."

